# On-Demand Tunability of Microphase Separation Structure of 3D Printing Material by Reversible Addition/Fragmentation Chain Transfer Polymerization

**DOI:** 10.3390/polym15173519

**Published:** 2023-08-23

**Authors:** Masaru Mukai, Mituki Sato, Wakana Miyadai, Shoji Maruo

**Affiliations:** 1 Graduate School of Engineering Science, Yokohama National University, 79-5 Tokiwadai, Hodogaya-ku, Yokohama 240-8501, Japan; 2 Faculty of Engineering, Yokohama National University, 79-5 Tokiwadai, Hodogaya-ku, Yokohama 240-8501, Japan; sato-mitsuki-gt@ynu.jp (M.S.); miyadai-wakana-hn@ynu.jp (W.M.)

**Keywords:** 3D printing, RAFT, stereolithography, heterogeneous 3D structures, polymerization-induced microphase separation

## Abstract

Controlling the phase-separated structure of polymer alloys is a promising method for tailoring the properties of polymers. However, controlling the morphology of phase-separated structures is challenging. Recently, phase-separated structures have been fabricated via 3D printing; however, only a few methods that enable on-demand control of phase separation have been reported. In this study, laser-scanning stereolithography, a vat photopolymerization method, is used to form a phase-separated structure via polymerization-induced microphase separation by varying the scanning speed and using macro-reversible addition/fragmentation chain transfer (macro-RAFT) agents with different average molar masses, along with multiarmed macro-RAFT agents; such structures were used to fabricate 3D-printed parts. Various phase-separated morphologies including sea-island and reverse sea-island were achieved by controlling the laser scanning speed and RAFT type. Heterogeneous structures with different material properties were also achieved by simply changing the laser scanning speed. As the deformation due to shrinkage in the process of cleaning 3D-printed parts depends on the laser scanning speed, shape correction was introduced to suppress the effect of shrinkage and obtain the desired shape.

## 1. Introduction

**Figure 1:**
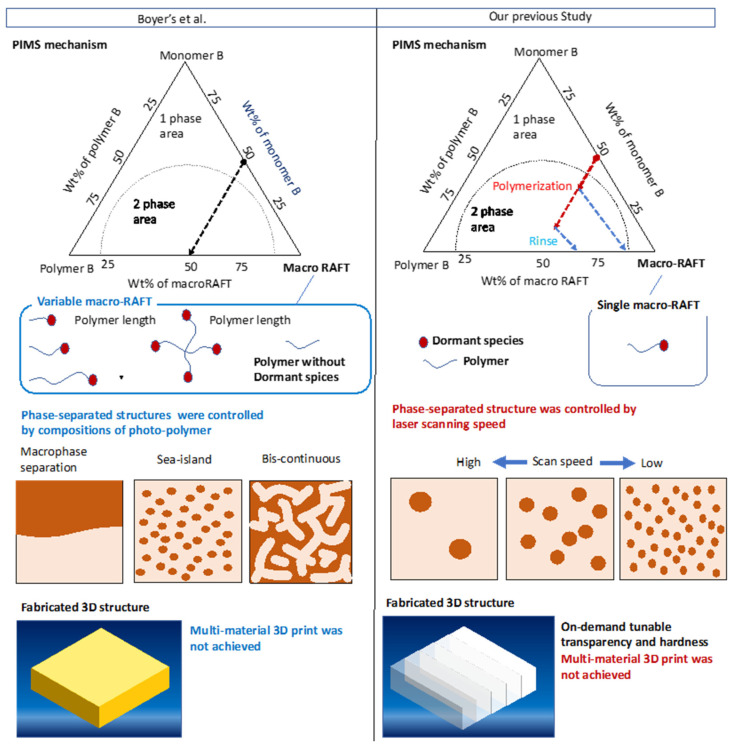
Comparison of our previous work with that of Boyer et al. [19,26,27] on stereolithography with RAFT polymerization via PIMS.

Stereolithography, a type of vat photopolymerization, is a 3D-printing technique that enables the high-precision fabrication of 3D objects [1,2] and is rapidly gaining popularity in medical [3,4,5,6], automotive [5,6], aerospace [5,6], and other fields [3,5,6]. The spread of this technology has heightened the demand for materials with various properties [5,7]. Numerous recent studies have developed stereolithography resins for use in reversible addition/fragmentation chain transfer (RAFT) polymerization [8]. These resins facilitate the secondary modification of 3D-printed objects, allowing for easy tuning of material properties. Ordinary radical polymerization eventually stops owing to the termination reaction. However, during photoinitiated RAFT polymerization, a dormant species is formed at the terminal of the polymer, allowing the polymer terminal to be reactivated by photo-irradiation [9]. This enables the polymerization of secondary polymers on the surface of a fabricated 3D object using RAFT polymerization [10,11]. Thus far, several stereolithography techniques using RAFT polymerization have been developed, including photoinduced electron transfer (PET) RAFT [11,12], photoinitiator RAFT [13], and photocationic RAFT [14]. These techniques have been applied in 4D printing [11,15], drug delivery systems [16], and self-healing materials [17,18]. Despite their apparent advantages, most RAFT techniques are limited to controlling the surface properties of 3D-printed models or cut surfaces. In contrast, Boyer et al. achieved the control of the material properties of a 3D-printed model, both on the surface of the 3D-printed model and inside the model, by photoinitiated polymerization-induced microphase separation (PIMS) [19]. PIMS is used to form phase-separated microstructures [20,21,22] and affords the control of the phase-separated structure of polymer alloys, thereby allowing the physical properties of polymeric materials to be tailored. The principle of PIMS can be explained using a triangular phase diagram and is summarized as follows [23,24]. Before polymerization, the mixture consists of a single phase containing a macro-RAFT agent, which has dormant species at the termini, and a monomer (Figure 1 left image, 1-phase area). The polymerization reaction is initiated, and the monomer is polymerized, after which the compatibility with the macro-RAFT agent decreases (2-phase area). Given that polymerization of the monomers occurs at the termini of the RAFT, a large phase-separated structure cannot be formed despite the reduction in compatibility; thus, a microphase-separated structure is formed. We also reported the use of PIMS for modifying the internal properties of a material [25]. The most significant difference between our previous study [25] and the study by Boyer et al. [19] is the parameters of the 3D printing processes that were varied to modify the material properties of the 3D-printed models. Boyer et al. produced phase-separated structures by adjusting the composition of the photocurable resin, such as the ratio of the macro-RAFT agents to monomer, the average molar mass of the macro-RAFT agents, and the branching of the macro-RAFT agents (multiarmed macro-RAFT agents) [19,26,27]. We achieved on-site control of the phase-separated structure during the 3D printing process by changing the local exposure dose at different scanning speeds [25] (Figure 1 right side). The degree of RAFT polymerization increases in proportion to the exposure time, which depends on the laser scanning speed of the laser-scanning stereolithography system. Adjusting the scanning speed induces a change in the shrinkage rate after rinsing. This shrinkage is believed to be caused by the dissolution of the uncured resin in the cleaning solution used to wash the 3D-printed model. The local degree of polymerization varies according to the scanning speed; areas fabricated with a high scanning speed contain more uncured monomers than those fabricated at a low scanning speed; thus, shrinkage during cleaning is greater in areas fabricated at a high scanning speed. Owing to this on-site control of the phase-separated structure, we achieved multi-material fabrication [28] with different material properties using a single resin in a single-step process by simply changing the scanning speed. Recently, Boyer et al. also reported a study on controlling the conversion rate of such macro-RAFT agents [26,27]. In their study, the conversion rate of macro RAFT agents was controlled by changing the irradiation time, not the laser scanning speed, because they used a stereolithography system based on digital light processing (DLP). However, they did not achieve multi-material fabrication in a single 3D printed model because DLP stereolithography systems employ simple two-dimensional image-based exposure and therefore do not allow local exposure control. 

In contrast, we constructed several types of lab-made stereolithography systems based on the laser-scanning method [29,30,31]. This method enables flexible control of the process parameters, such as the scanning speed, hatching distance, focus spot size, and laser wavelength. Furthermore, since The laser-scanning-based stereolithography method (SLA) generally has a higher resolution than DLP stereolithography [32]. Our approach is suitable for fabricating smaller devices such as 3D-printed printed electrodes [31], optical components [33], metamaterials [34], and micromachines [35,36]. However, the type of phase-separated structures obtained after 3D printing in our previous study was inferior to that of Boyer et al. (Figure 1) owing to differences in the composition of the photocurable resin. For example, the phase-separated structures observed in our study were sea-island structures, whereas Boyer et al. achieved not only sea-island structures (globular phase separation) but also bis-continuous, elongated, and macro-phase-separated structures. As the performance and functionality of polymer alloys depend on the phase structure, the lack of phase-separated structures is a drawback of our method.

**Figure 2:**
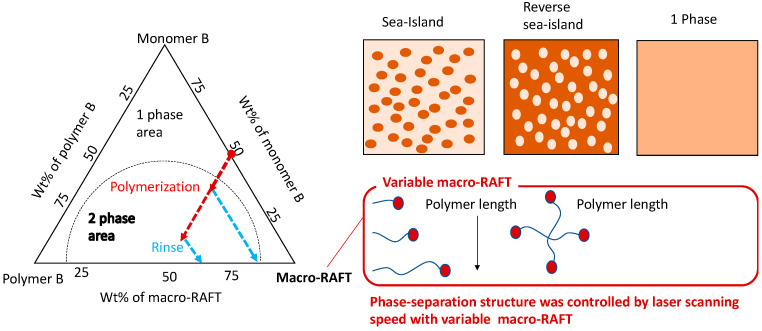
Schematic showing the control of phase-separated structures by varying the macro-RAFT agents and scanning speeds.

In this study, we synthesize macro-RAFT agents with various average molar masses and degrees of branching and evaluate the phase-separated structures obtained at different scanning speeds (Figure 2). The purpose of this research is to develop a stereolithography method that enables on-demand control of the phase-separated structures. In contrast with our previous study, which produced only the sea-island phase-separated structure, adjusting the scanning speed and type of macro-RAFT agents afforded the continuous phase, single phase, inverse sea-island, and other phase-separated structures. Notably, in order to realize high-precision multi-material 3D printing, shrinkage must be considered in advance. In this study, by modifying the original shape of a 3D model to correct for shrinkage, we obtain a 3D-printed object that is similar to the original design model.

## 2. Materials and Methods

### 2.1. Materials

Tetrahydrofuran (THF), ethanol, and sodium *N*,*N*-diethyldithiocarbamate trihydrate were obtained from FUJIFILM Wako Pure Chemical Corp. (Osaka, Japan). Styrene, benzyl diethyldithiocarbamate (benzyl-DTC), 2,2′-azobis(isobutyronitrile)(AIBN), tribromomethyl benzene, dibromo *p*-xylene, and *n*-butyl acrylate (BA) were purchased from Tokyo Chemical Industry Co., Ltd. (Tokyo, Japan). FDB-009 was obtained from YAMADA Chemical Co., Ltd. (Kyoto, Japan). Ethoxylated trimethylolpropane triacrylate (SR499) was purchased from TOMOE Engineering Co., Ltd. (Tokyo, Japan)

### 2.2. Characterization

Proton nuclear magnetic resonance (^1^H-NMR) spectra were acquired using a JNM-ECA500 spectrometer (500 MHz NMR, JEOL RESONANCE Inc., Tokyo, Japan). Size exclusion chromatography (SEC) was employed to determine the average molar mass using a Prominence Nano system (Shimadzu Corp., Kyoto, Japan) equipped with UV (SPD-20A) and refractive index detectors (RID-20A) and two columns (7.8 mm × 30 cm, 8 µm, G4000H and G3000H, TSKgel, Tosoh Corp., Tokyo, Japan). Scanning probe microscopy (SPM) was performed using an SPA400 (Seiko Instrument Inc., Chiba, Japan) apparatus, and the SPM phase-difference images were analyzed using the Gwyddion 2.58 instrument. The 3D models were observed using a VHX6000 microscope (Keyence Corp., Osaka, Japan). The width and length were measured from a top-view photograph of the fabricated object observed under the microscope. The height was measured from the side-view photograph of the fabricated object. Attenuated total reflectance Fourier-transform infrared (ATR-FTIR) data were acquired using an FT-IR6200 instrument equipped with an ATR PRO450-S (JASCO Corp., Tokyo, Japan).

### 2.3. Stereolithography Systems

**Figure 3:**
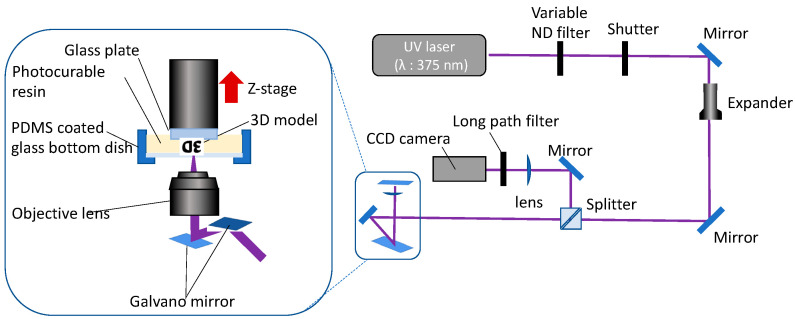
Schematic of a laboratory-made SLA stereolithography system.

A schematic of the stereolithography system is presented in Figure 3. This system comprises a semiconductor laser with an emission wavelength of 375 nm (DLD-XT 37550, LASOS Lasertechnik GmbH, Jena, Germany) that passes through a variable neutral density (ND) filter and an automatic shutter in order to adjust the laser power and activate/deactivate the laser, respectively. The laser diameter was expanded using a beam expander (magnification: ×10), and the laser light was directed toward a Galvano mirror (GM-1015, Canon Inc., Tokyo, Japan) after passing through a beam splitter cube. The light reflected by the Galvano mirror was focused on the boundary surface of the photocurable resin and the PDMS-coated glass palette using an objective lens with a numerical aperture of 0.1. The Galvano mirror was scanned in the horizontal plane in order to harden the shape of the cross-section, based on the slice data of the 3D model. The laser output used in the 3D fabrication was 40 mW after passing through a variable ND filter. The layer pitch and hatching distance were 30 and 3 µm, respectively. A charge-coupled device camera captured the laser light reflected by the glass palette, and a laser beam was used to adjust the fabrication start position. The 3D model was formed on a glass plate on which each layer was accumulated by pulling up the Z-stage. The glass plates were modified with methacrylate to improve the adhesion of the fabricated object. In contrast, the glass-bottom dish was coated with PDMS in order to improve the detachability from the fabricated object. The lab-made software automatically controlled the stages of fabrication of the 3D model using a 3D computer-aided design (CAD) model. The laser writing, accumulation, and variation of the scanning speed were controlled by the software.

### 2.4. Synthesis of Dithiocarbamate-Terminated Short-Length Poly(n-Butyl Acrylate) (ShortDTC-PBA)

**Scheme 1:**
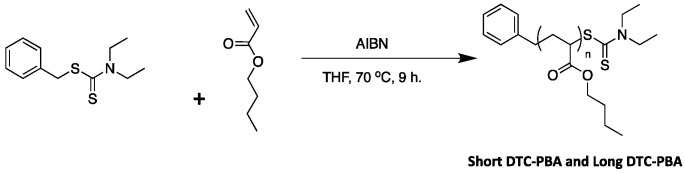
Synthesis scheme of ShortDTC-PBA and LongDTC-PBA.

ShortDTC-PBA was prepared by the RAFT polymerization of BA by heating (Scheme 1). BA (2.56 g, 20 mmol) was dissolved in THF (20 mL), after which AIBN (0.052 g, 0.3 mmol) and benzyl dithiocarbamate (DTC, 0.5 g, 2 mmol) were sequentially added to the polymerization tube. The solution was degassed using the freeze–pump–thaw method. The mixture was heated in an oil-bath at 70 °C for 12 h, after which the reaction was quenched in an ice bath (0 °C). The resulting polymer was purified by precipitation in cold methanol (0 °C). Prior reports pointed out that even under controlled radical polymerization such as RAFT, β-scission and short branching may occur at temperatures exceeding 70 °C [37,38]. Therefore, the synthesis temperature was kept below 70 °C in order to suppress the generation of macromonomers due to branching and β-scission. On the other hand, the multiarmed polymer was synthesized by the technique used for star-shaped polymers, as illustrated below. The short branching produced by side reactions is limited compared to that in the synthesized star-shaped polymers, and the polymer can be considered a linear polymer.

### 2.5. Synthesis of Dithiocarbamate-Terminated Long-Chain Poly(n-Butyl Acrylate) (LongDTC-PBA)

LongDTC-PBA was synthesized in the same manner as ShortDTC-PBA (Scheme 1), except that different quantities of BA (5.2 g, 40 mmol), THF (40 mL), AIBN (6.4 mg, 30 μmol), and benzyl-DTC (47 mg, 0.20 mmol) were used.

### 2.6. Synthesis of Bis-Functional RAFT Agent (BisDTC-Bz)

**Scheme 2:**
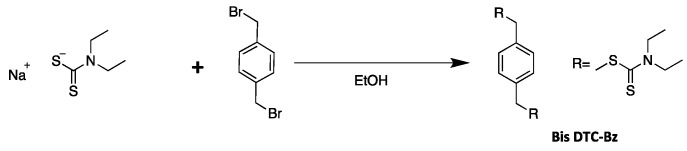
Synthesis scheme of BisDTC-Bz.

BisDTC-Bz was prepared using a method analogous to that reported by Grigoras et al. (Scheme 2) [39]. Sodium *N*,*N*-diethyldithiocarbamate trihydrate (2.56 g, 11.4 mmol) was dissolved in ethanol (40 mL) under argon atmosphere and cooled to 0 °C. Subsequently, a solution of dibromo-*p*-xylene (1 g, 3.8 mmol) in ethanol (10 mL) was added dropwise. After equilibration to room temperature (20–25 °C), the mixture was stirred for three days. The precipitate was filtered, and the solvent was removed. Recrystallization in ethanol yielded white, plate-like crystals.

### 2.7. Synthesis of Dithiocarbamate-Terminated 2-Arm Poly(n-Butyl Acrylate) (BisDTC-PBA)

**Scheme 3:**
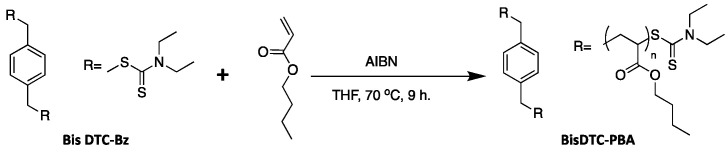
Synthesis scheme of BisDTC-BBA.

BisDTC-PBA was synthesized by a method analogous to that used to prepare ShortDTC-PBA (Scheme 3). BisDTC-Bz was used in place of benzyl-DTC as the RAFT agent, and the reaction was performed using BA (2.56 g, 20 mmol), THF (20 mL), AIBN (16 mg, 0.1 mmol), and BisDTC-Bz (100 mg, 0.25 mmol).

### 2.8. Synthesis of Tri-Functional RAFT Agent (TriDTC-Bz)

**Scheme 4:**
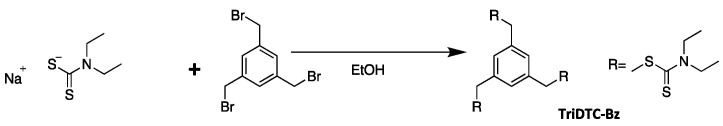
Synthesis scheme of TriDTC-Bz.

The synthesis of TriDTC-Bz was performed in accordance with the method of Grigoras et al. (Scheme 4) [39]. Sodium *N*,*N*-diethyldithiocarbamate trihydrate (2 g, 8.87 mmol) was dissolved in ethanol (40 mL) under argon atmosphere and cooled to 0 °C. A solution of 1, 3, 5-tribromomethyl benzene (1 g, 2.8 mmol) in ethanol (10 mL) was then added dropwise. After equilibration to room temperature (20–25 °C), the mixture was stirred for three days. The precipitate was filtered, and the solvent was removed. Recrystallization in ethanol yielded white, plate-like crystals.

### 2.9. Synthesis of Dithiocarbamate-Terminated 3-Arm Poly(n-Butyl Acrylate) (TriDTC-PBA)

**Scheme 5:**
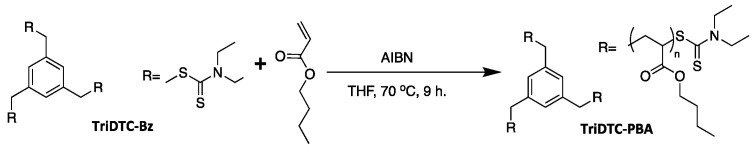
Synthesis scheme of TriDTC-PBA.

LongDTC-PBA was synthesized by a method analogous to that used to prepare ShortDTC-PBA (Scheme 5). TriDTC-Bz was used as the RAFT agent in place of benzyl-DTC, and the reaction was carried out with BA (2.56 g, 20 mmol), THF (20 mL), AIBN (16 mg, 0.1 mmol), and TriDTC-Bz (93 mg, 0.17 mmol).

### 2.10. Preparation of Photocurable Resin

The photocurable resin was prepared by mixing SR499 (1 g), macro-RAFT agents (1 g), ShortDTC-PBA, LongDTC-PBA, BisDTA-PBA (or TriDTA-PBA), and FDB-009 (0.5 mg) in a glass vial. FDB-009 is a light absorber that inhibits light penetration into the resin and limits the curing depth to prevent unintended excess curing. The samples were used without degassing.

## 3. Results and Discussion

### 3.1. Characterization of Dithiocarbamate-Terminated Macro-RAFT Agent

**Figure 4:**
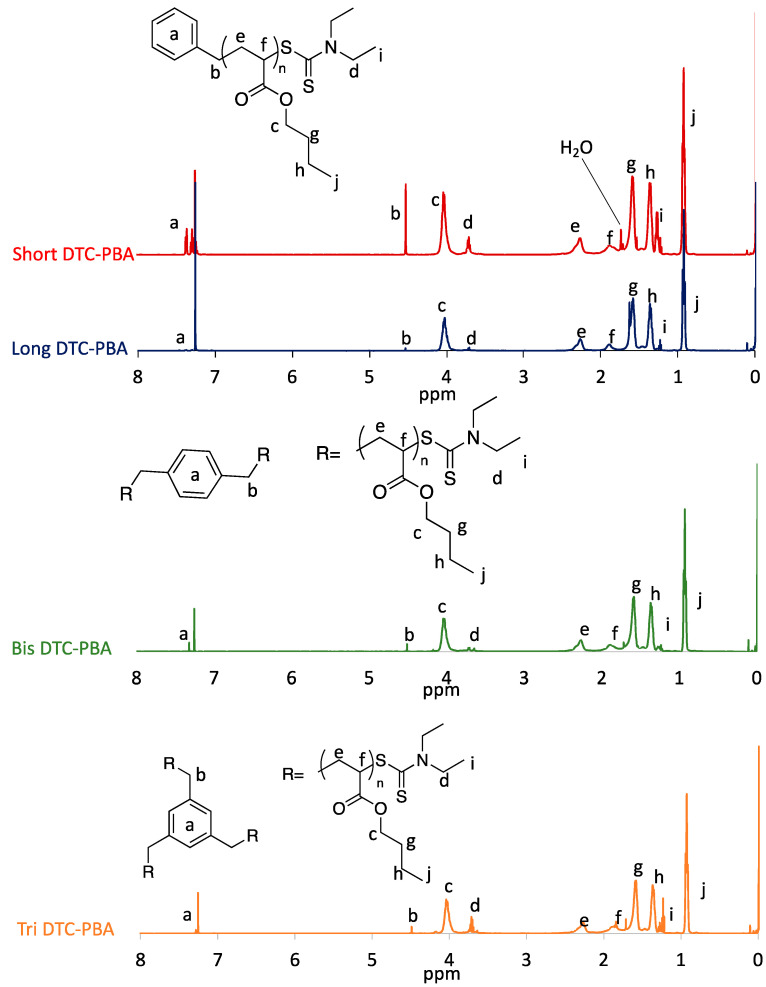
^1^H-NMR spectra of ShortDTC-PBA, LongDTC-PBA, BisDTC-PBA, and TriDTC-PBA in *d*-chloroform.

The proton (^1^H) NMR spectra of the synthesized macro-RAFT agents are shown in Figure 4. In all the spectra, peaks e and f at 2.27 and 1.86 originate from the main chain of poly(butyl acrylate) (PBA), whereas peaks c, g, h, and j, at 4.03, 1.59, 1.36 and 0.93 ppm, respectively, originate from the PBA side chain. The disappearance of the peaks derived from the acrylate group of BA (6.4, 6.1, and 5.8 ppm [40]) confirm the success of the polymerization reaction. The observed spectra are in good agreement with those of PBA reported in earlier studies [25,41], confirming the composition of the polymer. The peaks of the methyl group (peak j in Figure 4) and methylene group (peak d in Figure 4) of *N*,*N*-diethyldithiocarbamate (DTC), which acts as a dormant species, were also observed at 3.72 and 1.27 ppm, respectively, in all NMR spectra. DTC was assumed to be located at the termini of the linear polymer, and the approximate degree of polymerization (DP) was determined using Equation (1):

(1) $$DP=\frac{6}{Integrate\;value\;of\;peak\;i}$$

The j-peak was set to three (corresponding to three hydrogens) to calibrate the integrals in the calculation of the DP because peak j corresponds to the methyl group of PBA and the number of hydrogen atoms is three. Conversely, peak i, derived from the methyl groups of DTC, had an integral of six, corresponding to six hydrogen atoms. Therefore, the approximate DP can be calculated by dividing six by the integral value of peak i (Table 1). The molar ratios of the BA and RAFT agents were in good agreement with the DP of ShortDTC-PBA and LongDTC-PBA, suggesting that PBAs with different degrees of polymerization were synthesized as intended. In contrast, for BisDTC-PBA and TriDTC-PBA, the molar ratios of the BA to the RAFT agent exceeded the calculated DP because Equation (1) does not consider branching, and because BisDTC-PBA has two DTC groups, whereas TriDTC-PBA has three DTC groups. Accordingly, the calculated values should be doubled and tripled, respectively, to obtain good agreement with the molar ratio of the BA and RAFT agents. This suggests that multiarm polymers were obtained. Furthermore, peaks a and b are derived from aromatic alkyl groups. Multi-split peaks originating from the benzyl group were observed at 7.0–7.4 ppm in the spectra of ShortDTC-PBA and LongDTC-PBA. Integrals of the peaks corresponding to ShortDTC-PBA and LongDTC-PBA could not be obtained because the peaks overlapped with those of the deuterated chloroform solvent. In contrast, the spectra of BisDTC-PBA and TriDTC-PBA show singlets derived from aromatic hydrogen at 7.32 and 7.28 ppm, respectively. The difference in the positions of the peaks arising from these aromatic alkyl groups is due to the different RAFTs used in the synthesis. The integrals of peak a in the spectra of BisDTC-PBA and TriDTC-PBA are listed in Table 1. The i/a ratio indicates the ratio of the methyl group of DTC to hydrogen on the aromatic groups. The i/a ratios of 3 for BisDTC-PBA and 12 for TriDTC-PBA indicate that a multiarm polymer was obtained. The strong agreement between the i/a ratios determined from the structural formula and from the NMR spectra suggests that a multiarmed polymer was obtained.

Table 2 shows the number average molar mass (*M_n_*) and dispersity (*Đ_M_*) of the samples, determined by SEC. ShortDTC-PBA and LongDTC-PBA were identified as macromolecules with a *M_n_* of 2.1 k and 10.9 k, respectively. The *M_n_* and *Đ_M_* of the macro-RAFT agent used in our previous work (StandardDTC-PBA) were 5.0 k and 1.7, respectively. BA has a molar mass of 128.17 g/mol and its DP differs from the calculated value (Table 1). This is likely because the values obtained by SEC are not the absolute average molar mass, but are the converted average molar mass obtained using styrene as a reference material. ShortDTC-PBA and LongDTC-PBA were confirmed to be polymers with different molar masses. The *M_n_* values of BisDTC-PBA and TriDTC-PBA were 3.4 k and 3.6 k, respectively. The *M_n_* and molar ratio of the BA to RAFT agents in the macro-RAFT agents increased in the following order: ShortDTC-PBA, BisDTC-PBA, TriDTC-PBA, and LongDTC-PBA, suggesting that the DP can be controlled. The difference in the *M_n_* of BisDTC-PBA and TriDTC-PBA is smaller than the difference in the molar ratios of the BA and RAFT agents because SEC calculates the average molar mass based on the linear macromolecular exclusion volume and does not consider branching. This suggests that TriDTC-PBA is a multiarm polymer. This SEC data are also in good agreement with the NMR results.

### 3.2. Variation of Phase-Separated Structure with Macro-RAFT Agent and Scanning Speed

**Figure 5:**
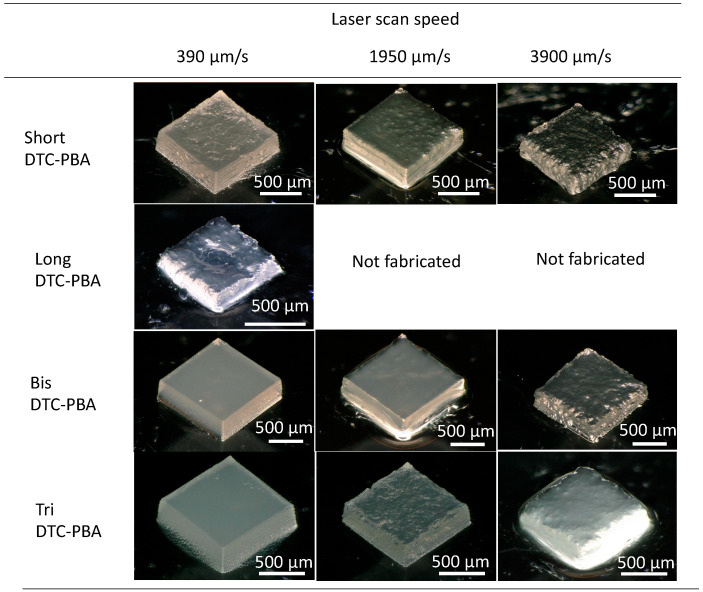
Bird’s-eye view of rectangular objects fabricated at different scanning speeds.

The fabrication of a rectangular object with dimensions of 300 × 1200 × 1200 µm using the synthesized macro-RAFT agents at different scanning speeds is depicted in Figure 5. Translucent-to-white solids were obtained under most conditions, although under certain conditions, the solids could not be modeled. For the same macro-RAFT agent, the solids fabricated with faster scanning speeds tended to have a higher transparency, more wrinkles on the surface, and less distinct contours. This is consistent with the results of our previous study [25]. Changing the optical and mechanical properties of ordinary light-cured resins without replacing the material is difficult [28]. Differences in the refractive index and phase-separated structure of SR499 and PBA result in different scattering behavior and thus differences in the transparency of the fabricated objects [25]. Our previous study and the paper by Boyer et al. also describe changes in the mechanical properties associated with changes in the phase-separated structure. Thus, it is reasonable that the mechanical properties also changed in this study [19,26,27]. The higher number of wrinkles formed at slower scanning speeds is thought to arise from shrinkage of the uncured resin, which is more extensive in objects fabricated at slower scanning speeds. This extensive shrinkage also results in an object with indistinct corners. Using ShortDTC-PBA, models were formed at scanning speeds of 390, 1950, and 3900 µm/s. In contrast, with LongDTC-PBA, the model was obtained only at a scanning speed of 390 µm/s. Using StandardDTC-PBA, moldings were fabricated at all scanning speeds in our previous study [25]. All photocurable resins were 1:1 (w/w) mixtures of monomers and macro-RAFT agents. A higher-*M_n_* macro-RAFT agent in a given polymer structure results in a lower concentration of dormant species in the photocurable resin. Therefore, LongDTC-PBA and ShortDTC-PBA were modeled at different percolation thresholds, even at the same scanning speed. With LongDTC-PBA, the cage effect may promote recombination and inhibit polymerization. In other words, LongDTC-PBA increases the likelihood of recombination without active polymerization because the polymer chains inhibit contact with the monomer even if radical species are generated. Multiarm macro-RAFT agents were fabricated at all three scanning speeds because the concentration of dormant species did not change as the number of arms increased.

**Figure 6:**
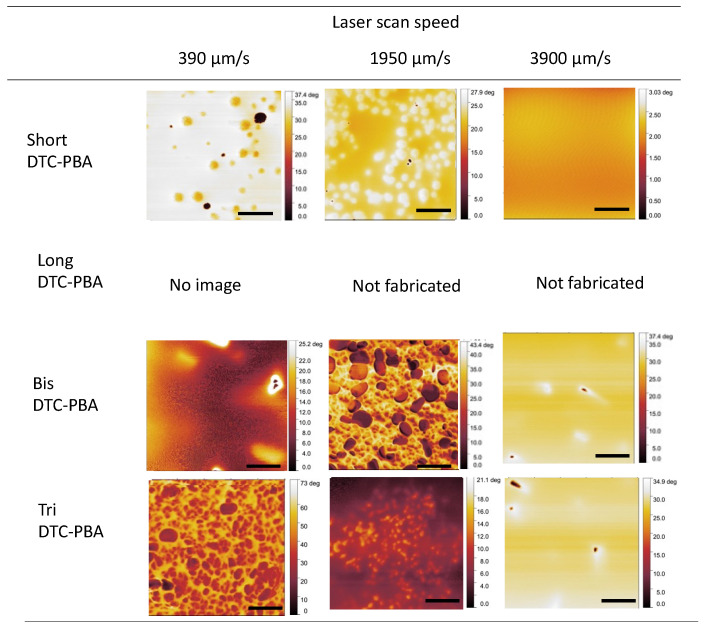
Phase-contrast image of models fabricated at different scanning speeds. Scale bar: 1 µm.

Figure 6 shows phase-contrast images of the rectangular objects fabricated at different scanning speeds; the phase-contrast provides a measure of the microscopic surface viscoelasticity [42]. Because PBA, the main component of the macro-RAFT agent, is a liquid, whereas polymeric SR499 is a solid, the darker-colored areas are rich in highly viscoelastic polymeric SR499, whereas the lighter-colored areas are rich in PBA. When fabricated with the StandardDTC-PBA described in our previous report [24], a sea-island structure consisting of darker-colored islands and lighter-colored seas (island-sea structure) was observed for the samples modeled at scanning speeds of 390, 1950, and 3900 µm/s. In addition, the area occupied by the islands tended to increase as the scanning speed decreased. In the phase-contrast images obtained with ShortDTC-PBA, phase-separated structures with different contrasts were observed at scanning speeds of 390 and 1950 µm/s, whereas no phase separation was observed in the samples fabricated at a scanning speed of 3900 µm/s. The resulting object is considered to be located in the one-phase region of the triangular phase diagram in Figure 1. The model fabricated at 3900 µm/s exhibited higher transparency than the samples fabricated at 390 and 1950 µm/s (Figure 5), resulting in relatively transparent fabricated moldings without scattering based on the phase-separated structures. In other words, a relatively transparent cured material is formed because there is no difference in the refractive indices of the components. Phase separation was not observed in the samples fabricated with ShortDTC-PBA at 3900 μm because ShortDTC-PBA is a macro-RAFT agent with a smaller *M_n_* than StandardDTC-PBA, is more compatible than StandardDTC-PBA, and exists in a single-phase region. (The Flory-Huggins theory indicates that shorter polymers undergo a greater entropy change upon admixture, compared to longer polymers.) Moreover, local polymerization was limited (insufficient) at this high scanning speed. When ShortDTC-PBA was used at 390 µm/s, a sea-island structure was observed, where the islands appeared as dark-colored areas, as observed with StandardDTC-PBA. In contrast, the use of ShortDTC-PBA at 1950 µm/s resulted in a reverse sea-island structure with the islands appearing as light-colored areas. In our previous study, only sea-island structures were observed, whereas herein, sea-islands, inverse sea-islands, and homogeneous structures were created using macro-RAFT agents with a low *M_n_* by adjusting the scanning speed. These results suggest that on-demand control of the phase separation is achieved dependent on the scanning speed and macro-RAFT agent type. As shown in Figure 5, Only the sample formed with LongDTC-PBA at 360 µm/s was able to adopt a shape (Figure 5). However, a phase-contrast image could not be obtained even though the cantilever was changed from SI-DF20 (127 kHz,18 N/m) to SI-DF3P2 (70 kHz, 2.1 N/m) with a low spring constant. This suggests that the object is soft, which is consistent with the fact that it could not be formed at high scanning speeds. The models fabricated using BisDTC-PBA and TriDTC-PBA at 3900 m/s did not possess clear phase-separated structures, consistent with the fact that a relatively transparent model was obtained (Figure 5) for the same reason as stated for ShortDTC-PBA. Fabrication using BisDTC-PBA at a scanning speed of 1950 µm/s resulted in dense sea-island structures, whereas TriDTC-PBA formed inverted sea-island structures under the same conditions. Further slowing the scanning speed to 390 µm/s produced continuous phase separation in the case of BisDTC-PBA, whereas a dense sea-island structure was obtained with TriDTC-PBA. The multiarm macro-RAFT agents formed denser phase-separated structures at 1950 and 390 µm/s than those obtained with ShortDTC-PBA and StandardDTC-PBA. Linear macro-RAFT agents with longer molecular chains have poorer compatibility than shorter molecular chain and are more prone to phase separation. In contrast, linear macro-RAFT agents with longer molecular chains have a lower concentration of dormant species, resulting in lower secondary polymerization than in linear macro-RAFT agents with lower degrees of polymerization, assuming the same exposure time. The compatibility of the multiarm macro-RAFT agents decreased as the number of arms increased. In this case, the concentration of dormant species in the system remained constant even as the number of arms increased, and the polymerization efficiency remained constant with an increase in the number of arms, which is thought to result in a denser phase-separated structure.

### 3.3. Shrinkage and Correction of Fabricated Objects

**Figure 7:**
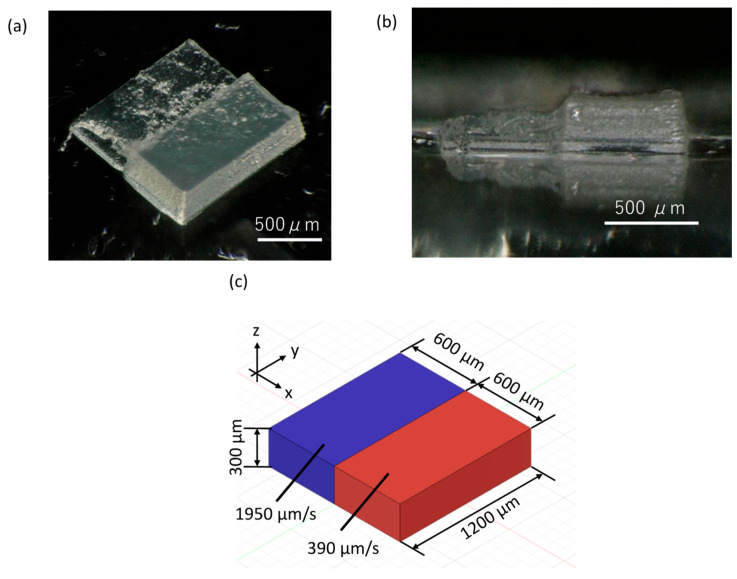
(**a**) Bird’s-eye view, (**b**) side-view, and (**c**) 3D-CAD image of models fabricated at different scanning speeds.

In our previous study, the number of stacked cross-sectional shapes was manually adjusted to compensate for the discrepancy between the designed and fabricated objects to mitigate shrinkage and obtain objects as similar as possible to the envisioned design [24]. However, shrinkage occurs in all three dimensions, making it difficult to fabricate complex 3D structures. As a first step to address this issue, a simple strip model was fabricated at two different scanning speeds, and the correction model was re-designed and applied by estimating the shrinkage rate along the three axes. An experimental model was developed in which two rectangular objects with dimensions of 300 × 600 × 1200 µm were fabricated at scanning speeds of 1950 µm/s and 390 µm/s. The photocurable resin consisted of a 1:1 mixture of StandardDTC-PBA and the monomer. This model confirmed that the rectangles fabricated at 1950 µm/s experienced more extensive shrinkage than those fabricated at 390 µm/s (Figure 7).

The dimensions of the designed and fabricated rectangular samples are listed in Table 3 along with the shrinkage rates. Measurements of the length and width were taken on the top surface of the fabricated object, and the height was measured from the side. The dimensions of the object fabricated at 1950 µm/s are smaller than those of the object fabricated at 390 µm/s, indicating significant shrinkage. This is consistent with our previous report on the shrinkage mechanism; however, shrinkage anisotropy was observed, with greater shrinkage along the width and height directions than along the length direction, because the substrate and fabricated object both regulate the shrinkage, resulting in less shrinkage in the longitudinal direction.

**Figure 8:**
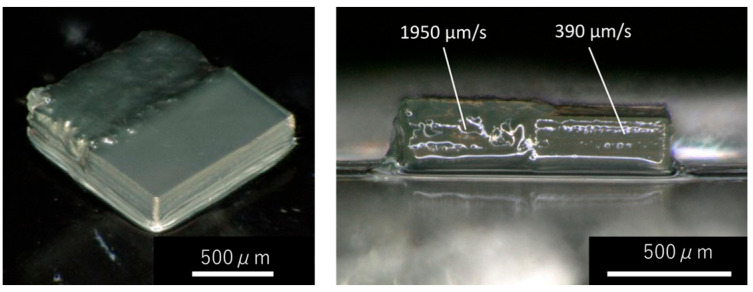
Bird’s-eye and side-views of models fabricated at different scanning speeds with shrinkage compensation.

Models were created using the corrections based on shrinkage. The fabricated object with shrinkage compensation was modeled three times to obtain the mean and standard deviation of the dimensions of the object. No correction was made to the length because no significant shrinkage was observed along this direction; however, corrections were made to the width and height directions, which were significantly affected by shrinkage. The areas near the substrate were corrected in an inverted trapezoidal shape as shrinkage was suppressed by the substrate. The correction factors and examples of fabrication with the corrected models are presented in Table 4 (upper column) and Figure 8, respectively. Wrinkles due to shrinkage were observed in the area fabricated at 1950 µm, whereas the area fabricated at 390 µm/s was at the same height of fabrication object (Figure 8). Table 4 (lower column) shows that the shrinkage correction was 90% effective in adjusting the heights, lengths, and widths. Additionally, the height error was related to the length and width, likely because the ‘origin’ was manually adjusted before fabrication, which tended to cause large variations in the height direction. Regardless of the nearly identical height, the area fabricated at 390 µm/s appeared whiter and the area modeled at 1950 µm/s appeared translucent. This is in good agreement with the results of our previous study, which suggests that the transparency of the fabricated object is dependent on the phase-separated structure.

**Figure 9:**
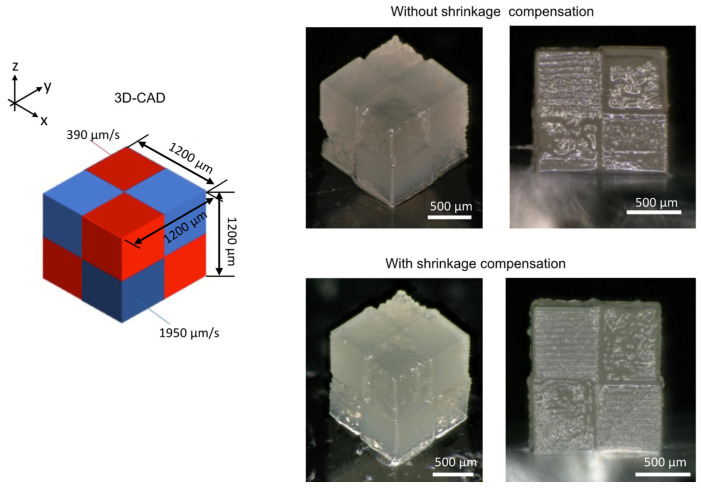
Fabricated model of checkered cube with and without shrinkage compensation.

To demonstrate the effectiveness of the shrinkage correction in more complex structures, checkered-cube structures fabricated with and without shrinkage correction were compared (Figure 9). In this case, the width and length were assumed to shrink isotropically and a shrinkage correction was applied. The shrinkage of the checkered cubes built without shrinkage correction at 50 µm/s was 84, 83, and 93% along the width, length, and height, respectively. Conversely, the shrinkage area of the checkered cube fabricated with shrinkage correction at 50 µm/s was 99%, 99%, and 123% along the width, length, and height, respectively. The shrinkage in width and length were greatly improved, whereas the shrinkage in height was overcompensated, likely as a result of errors in focusing before modeling, complex interactions between regions with different scanning speeds owing to the more complex structure modeled, and the fact that the checkered-cube model is nearly three times as tall (1200 µm) as the model for which shrinkage was calculated (300 µm), which magnifies the shrinkage error. Although the height direction was larger than that in the model, the heights of the two regions with different scanning speeds were aligned, and the accuracy of the xy-plane was significantly improved. Checkered cubes were also fabricated in our previous report [24], but with scanning speeds of 975 µm/s and 195 µm/s, where slower scanning speeds than those used in this study were employed to reduce shrinkage. Despite the increase in the scanning speed, the dimensions of the designed and actual model matched, confirming the effectiveness of the correction. To achieve a more accurate correction, the distortion caused by the interaction between objects at different scanning speeds must be resolved, which requires methods such as the finite element method (FEM) to eliminate distortions caused by the interaction between objects with different mechanical properties. The material properties may differ due to differences in the adhesion between layers and between different materials when molding with RAFT agents and using ordinary photoinitiators such as diphenyl(2,4,6-trimethylbenzoyl)phosphine oxide (TPO). This is because adhesion between materials using RAFT agents will result not only from co-polymerization of the unreacted polymerizable functional groups in the cured material with the material during curing, but also from polymerization initiated by dormant species present in the vicinity of the cured material.

## 4. Conclusions

Macro-RAFT agents with different average molar masses and multiarm macro-RAFT agents were synthesized, mixed with the monomer SR499, and used to fabricate models at different scanning speeds via SLA stereolithography. For the same macro-RAFT agent, lower scanning speeds tended to produce whiter and more clearly defined structures. SPM phase-contrast images revealed the presence of multiple phase-separated structures, such as sea-island, inverse sea-island, uniform, and continuous structures, in the fabricated model owing to changes in the macro-RAFT agent and scanning speed, whereas our previously reported approach resulted in only sea-island structures. These results indicate that the on-demand control of phase separation is achieved depending on the scanning speed and macro-RAFT agent type. Such control of phase-separated structures will contribute to the production of multimaterial structures with tailored properties. Automated shrinkage compensation in multimaterial 3D printing was also achieved. Consequently, the designed structure can be realized by shrinkage compensation even if the original 3D object is complex in the xy-plane. Shrinkage compensation in multimaterial 3D printing will be useful for high-precision 3D printing because shrinkage and swelling can occur after printing owing to several factors, including temperature changes, degreasing operations, and washing.

## Figures and Tables

**Table 1:** Integrals obtained from the ^1^H-NMR spectra ^a^.

Sample	Peaks		DP ^b^	[BA]/ [RAFT]	i/a
	a	i			
ShortDTC-PBA	- ^c^	0.67	9	10	-
StandardDTC-PBA ^d^	- ^c^	0.14	41	50	-
LongDTC-PBA	- ^c^	0.0025	240	200	-
BisDTC-PBA	0.029	0.091	66	80	3.1
TriDTC-PBA	0.012	0.150	40	118	12.5

^a^ To calibrate the integral, j peaks were set to 3H. ^b^ Estimated DP. ^c^ Overlap with CHCl_3_ peak. ^d^ Our previously reported macro-RAFT agent.

**Table 2:** *M_n_* and *Đ_M_* of synthesized Macro-RAFT agents measured by SEC ^a^.

Sample	*M_n_*	*Đ_M_*
ShortDTC-PBA	2.1 k	2.2
StandardDTC-PBA	5.0 k	1.7
LongDTC-PBA	10.9 k	1.6
BisDTC-PBA	3.4 k	1.8
TriDTC-PBA	3.6 k	1.7

^a^ SEC (THF, polystyrene standard).

**Table 3:** Design and actual rectangular dimensions and shrinkage rates.

Scan Speed (µm/s)	Dimension of 3D CAD Model		
	X: Width (µm)	Y: Length (µm)	Z: Height (µm)
1950	600	1200	300
390	600	1200	300
Scan speed (µm/s)	Measured dimensions of fabricated objects		
	X: Width	Y: Length	Z: Height
1950	467 µm (79%)	1117 µm (93%)	215 µm (71%)
390	569 µm (94%)	1148 µm (96%)	282 µm (94%)

**Table 4:** Rectangular dimensions and shrinkage rates of the designed and fabricated objects with shrinkage compensation.

Scan Speed (µm/s)	Dimensions of the 3D CAD Model with Compensation Values		
	X: Width (µm)	Y: Length (µm)	Z: Height (µm)
1950	771	1200	420
390	663	1200	330
Scan speed (µm/s)	Measured dimensions of fabricated objects		
	X: Width	Y: Length	Z: Height
1950	558 ± 7 µm (93 ± 4%)	1158 ± 5 µm (96 ± 0.4%)	309 ± 11 µm (103 ± 4%)
390	598 ± 16 µm (100 ± 2%)	1187 ± 4 µm (99 ± 0.3%)	284 ± 19 µm (93 ± 6%)

## Data Availability

The datasets generated and/or analyzed during the current study are available from the corresponding author on reasonable request.
